# The perception of pediatric sickle cell anemia patient's caregivers toward hematopoietic stem cell transplantation (single-center experience, Saudi Arabia)

**DOI:** 10.3389/fped.2023.1205351

**Published:** 2023-05-23

**Authors:** Dania A. Monagel, Naglla Alemam, Manar Betar, Fay N. Alnafisi, Dania E. Faidah, Reema E. Aloteibi, Muhammad Khan, Israa A. Malli

**Affiliations:** ^1^ College of Medicine, King Saud bin Abdul-Aziz University for Health Sciences, Jeddah, Saudi Arabia; ^2^ King Abdullah International Medical Research Center, Jeddah, Saudi Arabia; ^3^ Department of Oncology, Ministry of the National Guard-Health Affairs, Jeddah, Saudi Arabia

**Keywords:** sickle cell disease, pediatric, perception, transplant, parent, caregiver

## Abstract

**Background:**

Sickle cell disease (SCD) is a relatively common genetic disorder in Saudi Arabia characterized by the predominance of sickle hemoglobin (HbS). Although multiple supportive care options exist for patients with SCD, hematopoietic stem cell transplantation (HSCT) is the only cure available and has become highly successful, with an almost 91% overall survival rate. However, pursuing this procedure is still restrained as a curative treatment option. Therefore, this study aimed to evaluate the perception of parents' caregivers at the National Guard Hospital pediatric hematology clinic regarding using HSCT as a curative approach for their children with SCD.

**Methods:**

This is a cross-sectional study of the interviewer-administered survey distributed utilizing electronic devices to caregivers of pediatric patients with SCD. Subjects were recruited from Pediatric Hematology & Oncology clinics at National Guard Hospital Affairs in King Abdulaziz Medical City, Jeddah, Saudi Arabia. An estimated sample size of 100 was initially calculated out of 140 pediatric SCD patients; 72 responses were collected from participants. All study participants gave informed consent. All results were analyzed using SPSS; moreover, statistics were set at a CI of 95% and a *p* < 0.05. In addition, inferential and descriptive statistics were done.

**Results:**

Of all respondents, 42 (67.8%) would accept HSCT if their hematologist recommended it. However, approximately 7 (11.3%) were not interested in the procedure, and the rest, 13 (21%), were uncertain. The most reported reasons for HSCT rejection among all respondents were attributed to side effects 31 (50.8%), lack of knowledge 8 (13.1%), and misconception toward the procedure 22 (36.1%).

**Conclusion:**

The results of this study were consistent with the fact that most caregivers would follow along with HSCT if it seemed to be fit and was recommended by their hematologists. However, to the best of our knowledge, our study being the first of its kind in the region, further research in the kingdom on the perception of HSCT is needed. Nonetheless, further patient education, an increase in caregivers' knowledge, and enlightenment of the medical team on HSCT as a curative option for sickle cell disease are vital.

## Background

Sickle cell disease (SCD) is a group of inherited hemoglobin disorders characterized by the preponderance of sickle hemoglobin (HbS) (1). HbS is formed due to a change in DNA codon wherein Valine, an amino acid, is substituted for Glutamate at amino acid 7 of the beta-globin chain (2). Due to this genetic alteration, several factors can cause the polymerization of HbS, which will affect the shape of the RBC and change it into a sickle cell (3). The hallmark of SCD is vaso-occlusion. Consequently, patients usually present with variable clinical courses characterized by recurrent acute crises with a stage of clinical stability. Those crises are often unpredictable but must be managed promptly, which poses a significant burden on caregivers, particularly in a resource-limited setting (4).

There are multiple supportive care options for SCD patients, such as red cell transfusion, hydroxyurea, and lately approved disease-modifying therapies (crizanlizumab, L-glutamine, voxeltor), yet none of them is a definitive treatment (5). Hematopoietic stem cell transplantation (HSCT) has proven its efficacy as a curative option with a greater than 91% overall survival (6). In the recent decade, HSCT has become a highly successful and applicable procedure worldwide. Our local experience has shown acceptable transplant-related morbidity and mortality (7). Despite that advancement in HSCT, barriers still exist that prevent its utilization which include; the risk of short and long-term effects, lack of HLA-matched sibling donors, uncertainty voiced by the clinician as well as patients, access to health care, social/cultural factors and knowledge/awareness (8).

Moreover, there is a new promising transformative treatment for SCD based on gene therapy, a gene addition product (Betibeglogene autotemcel) approved in Europe for some forms of thalassemia and being investigated for other types of thalassemia and SCD (9). Recently Frangoul et al. reported a successful increase in fetal hemoglobin, transfusion independency, and elimination of vaso-occlusive crisis in a patient with SCD using CRISPR-Cas9 gene editing through BCL11A erythroid-specific enhancer (10). Although the result seems promising, long-term follow-up is required to assess the sustainability of this positive effect and the risk of using myeloablation. In addition to the cost-effectiveness when compared to HSCT.

Around 100 million individual patients globally suffer from SCD, with 300,000 new births annually (4, 11). SCD is a relatively common genetic disorder in Saudi Arabia. Based on the premarital screening, sickle cell gene prevalence was estimated to be 0.26%, with the highest in the Eastern Province being 1.2%. Though, premarital screening could underestimate the true prevalence of SCD since it only depends on the survival of the patients with SCD up to the age of marriage. A regional experience with newborn screening in the Eastern Province found that the sickle cell trait prevalence is 21% and 2.6% for SCD (12). AL-Sultan et al. had estimated that approximately 61,000 patients with SCD reside in Saudi Arabia and around 10,536 patients are HSCT candidates (of which 2,148 are children) (13).

Previous literature has shown that SCD considerably impacts the patients' and their families' quality of life (QoL) since it is a life-long event (14). Several aspects of the patient's life are usually affected, which is usually linked to the disease severity. SCD can lead to a higher rate of school absenteeism, social vulnerability, low income, unemployment, and psychological issues. The negative impacts usually extend to the caregivers and siblings, triggering emotional disturbance, abnormal family dynamics, relationship struggles, and lack of personal development (4, 14). Madani et al. reported on QoL among caregivers of children with SCD in the Western region of Saudi Arabia; they showed that negative emotions were more often among mothers. Sleep quality and sexual life were the most disturbed aspects, while social connection, cognitive abilities, and career achievement were relatively maintained (15).

Today, the public's knowledge of the matter has relatively improved. However, their knowledge remains insufficient, leading to a restriction on donation and acceptance of the HSCT as a treatment option for SCD (16). Therefore, this study aimed to further increase our knowledge of the percentage of pediatric sickle-cell anemia patients' caregivers that decide upon choosing HSCT as a curative option for their children. Moreover, our study sheds light on the general misconceptions' parents might believe to be true regarding HSCT. Lack of awareness, limited availability of transplant teams, limited logistic support, and other countable reasons usually redirect them to look for alternative treatments, although curing SCD is made possible with HSCT (16).

## Methods

This study was carried out using a cross-sectional survey-based design. This design was chosen to evaluate the perception and attitudes of sickle cell disease (SCD) patients' caregivers toward HSCT as a curative treatment. The families of SCD patients were informed about the study purpose, voluntary participation, and confidentiality of the research data. Informed consent was obtained from all the participants. The study was approved by Institutional Review Board (King Abdullah International Medical Research Center, KAIMRC). It was started on January 2020 for 12 months and was conducted at the Pediatric Hematology clinic (King Abdul-Aziz Medical City, Jeddah, a tertiary center in Saudi Arabia). Moreover, the study population for this research included families of SCD patients who attend the National Guard Health Affairs (NGHA) hematology clinic between 0 months and 14 years of age. This survey was conducted through a non-probability consecutive sampling technique.

### Study survey

The data used for the study was collected from the patient's caregivers. They were surveyed utilizing an online questionnaire (30 items) provided via an electronic tablet device distributed by trained medical personnel (who also ensured the caregiver's understanding of the questions and answers). The survey was divided into four subsections to measure each study objective. Questions included were of multiple-choice type, and the Likert scale was also used. The first section of the study collected demographical information such as the relationship between the caregiver and the patient, gender, age, type of sickle cell disease, and education level. The second section of the survey covered the caregivers' perception of HSCT, and the questions measured their concept and attitude toward HSCT. The third section of the survey included questions corresponding to the study respondents' misconceptions about HSCT. In contrast, the fourth section of the survey included questions corresponding to restrictions and barriers preventing caregivers from choosing HCST, such as procedures, disease-related complications, or failure to find eligible donors.

The development of the questionnaire was done through an extensive literature review. Retrieved questions were taken from multiple sources after modifying them according to the study goals and were then translated into Arabic (17). The study questionnaire was developed in English and Arabic by bilingual experts. For validation, three subject experts assessed the content validity of the study tool, and a pilot survey was distributed to 70 participants from the general population sent online via social networking applications. The questions were adjusted based on the respondents' feedback. The study tool's internal consistency was determined by using the reliability coefficient Cronbach's alpha (*α* = 0.87). The time to complete out survey form was approximately 15–20 min.

### Estimated sample size

Among HCST candidates that live in Saudi Arabia with SCD, 20% are children (13). Our clinic has around 140 children with SCD. Using an online sample size calculator (RaoSofi website), an estimated sample of 90 responses are needed with a confidence level of 95% and a 5% margin of error. Before survey distribution, the survey response rate was set to be at least 60% of the estimated sample size (18).

### Data analysis

This survey-based study is cross-sectional, in which a 140 pediatric SCD patients were included; 72 responses were collected from participants (80% response rate of the survey). The significance level for all statistical tests was 2-sided *p* < 0.05. Statistical analysis was performed using the (SPSS), IBM Corp. Released 2011. IBM SPSS Statistics for Windows, Version 20.0. Armonk, NY: IBM Corp. Numerical variables were presented as median and range. Categorical variables, like gender, educational level, and marital status, were presented by frequency and percentage. Chi-square or Fisher exact was used to compare two categorical variables where appropriate.

## Results

### Child and parents demographics

Seventy-two caregivers of children with SCD completed the survey (10 of them did not answer all the questions as some were not applicable, and they did not proceed to them). Subjects ages 11 months to 14 years were restrictively represented. The median age of caregivers was 38 years; conversely, the median age of children with SCD was 8 years. Sixty-six (91.7%) of caregiver respondents were the patients' parents. Most of the respondents were high school graduates, 28 (38.9%). The marital status of the patient's caregivers varied between married 64 (88.9%), single 6 (8.3%), and divorced 2 (5.8%) (Table 1).

**Table 1:** SCD patients and their caregivers’ demographics.

	*n* = 72	%
Parent gender		
Female	47	65.3
Male	25	34.7
Parent age		
Birth-20 years	3	4.2
21–30 years	50	69.4
>30 years	19	26.4
Parent education		
Elementary	7	9.7
Middle school	11	15.3
High school	28	38.9
College undergraduate	24	33.3
College postgraduate	2	2.8
Marital status		
Single	6	8.3
Married	64	88.9
Divorced	2	5.8
Occupation		
Student	10	13.9
Employed	31	43.1
Retired	2	2.8
Unemployed	24	33.3
House wife	5	6.9
Caregiver relation to child		
Parents	66	91.7
Siblings	6	8.3
Child gender		
Female	31	43.1
Male	41	56.9

### Perception of HSCT

Structured survey tools investigated the knowledge of pediatric SCD patients' caregivers of HSCT. Fifty-nine (81.9%) of the respondents had previous knowledge about HSCT. However, there was a variety in their source of expertise, in which doctors (63%) and social media and internet (16.7%) were the highest. Although 40 (55.6%) were not previously offered the HLA typing procedure, 36 (58.1%) understood the meaning of sibling-HLA match, and 50 (80.6%) had further interest in learning about HSCT. Seventy-nine percent of the respondents believe the survival rate is around 80%–100%. On the contrary, among several caregivers, 62.1% agreed that the mortality rate associated with HSCT is 5%–10% (Table 2).

**Table 2:** Association of parents’ knowledge and acceptance likelihood.

	Parent likelihood to approve HCT procedure			*p*
	No	Maybe	Yes	
	*n* = 7	*n* = 13	*n* = 42	
Parent perception of mortality rate with HSCT				**0.739**
0%	0	3	12	
5%–10%	5	6	18	
11%–30%	1	3	8	
>30%	1	1	4	
Parent perception of survival rate with HSCT				**0.238**
<80%	5	9	19	
>80%	2	4	23	
Parent prior knowledge of sibling HLA match^a^				**0.556**
A	1	2	12	
B	0	0	0	
C	5	10	21	
D	1	1	9	

Fisher exact test.

^a^ A: A patient with sickle cell disease donates stem cells to a brother or sister. B: A patient with sickle cell disease donates stem cells to another person with sickle cell disease. C: A patient with sickle cell disease gets healthy stem cells from a brother or sister to replace abnormal blood cells in the patient's body. D: A patient with sickle cell disease gets healthy stem cells from someone not related to them to replace abnormal blood cells in the patient's body.

### Interest in HSCT as a cure for SCD

Approximately 42 (67.8%) caregivers would accept HSCT if their doctor recommended it. However, around 7 (11.3%) were not interested in the procedure, and the rest, 13 (21%), were uncertain. The most reported reasons for HSCT refusal among all respondents (even for those who might accept the procedure) were attributed to lack of knowledge 8 (13.1%), side effects 31 (50.8%), and misconception toward the procedure 22 (36.1%).

The possible side effects that they are mainly concerned about were 45 (61.6%) death, followed by malignancy 30 (41.1%), risk of GVHD 18 (24.7%), and lastly, treatment-related infertility 12 (16.7%) of caregivers.

### Patient-related characteristics associated with increased interest in HSCT

Interest in transplantation was associated with the severity of the disease and the quality of life. Each SCD patient experienced at least one complication, including pain crisis (66.7%), acute chest syndrome (ACS) (33.3%), splenic sequestration (33.3%), delayed puberty or reduced growth (30.6%), lung complications (26.4%), osteomyelitis (20.8%), cholecystectomy or splenectomy (20.8%), bone necrosis (17.8%), transcranial doppler abnormality (TCD) (11.1%), stroke (9.7%), chronic ulcer (6.9%), cardiac issues (5.6%), liver issues (4.2%) and kidney issues (2.8%). Table 3 shows a trend toward acceptance from the parents if the child has more complications, although statistically not significant.

**Table 3:** Association between the SCD complications and the acceptance rates of HCCT.

	HSCT				*p*
	No		Yes		
	*n* = 20	%	*n* = 42	%	
Bone necrosis					**0.907** ^a^
Yes	4	36.4	7	63.6	
No	14	31.1	31	68.9	
I don't know	2	33.3	4	66.7	
Stroke					**0.842** ^a^
Yes	1	16.7	5	83.3	
No	18	34.0	35	66.0	
I don't know	1	33.3	2	66.7	
Acute chest syndrome					**0.335** ^a^
Yes	9	40.9	13	59.1	
No	10	26.3	28	73.7	
I don't know	1	50.0	1	50.0	
Pain crisis					**0.768** ^b^
Yes	15	34.1	29	65.9	
No	5	27.8	13	72.2	
Splenic sequestration					**0.184** ^a^
Yes	6	26.1	17	73.9	
No	11	31.4	24	68.6	
I don't know	3	75.0	1	25.0	

^a^ Fisher exact test.

^b^ Chi-squared test.

For most patients, 24 (33.3%) had 1–2 crises per year, whereas 3 (4.2%) had 5–6 crises yearly (Table 4). Moreover, caregivers with more than one child with SCD and parents who have had to miss days of work each year secondary to their offspring's disease were more willing to accept HSCT. In addition, parents whose children were treated by blood exchange transfusion were interested in accepting the HSCT. Few caregivers (15.4%) believe that SCD will get worse over time, (53.7%) have optimistic predictions that it will get better, and (30.9%) think that the condition of their offspring will remain the same.

**Table 4:** Association between the number of crises, parent's and child's absence days with the acceptance rates of HSCT.

	HSCT				*p*
	No		Yes		
	*n* = 20	%	*n* = 42	%	
Crises					**0** **.** **603**
No	6	37.5	10	62.5	
Yes	14	30.4	32	69.6	
Child absence days					**0.046**
No	6	20.0	24	80.0	
Yes	14	43.8	18	56.3	
Parent absence days					**0.713**
No	11	34.4	21	65.6	
Yes	9	30.0	21	70.0	

Chi-squared test.

## Discussion

This study aimed to provide a representation of regional caregivers' perception of children with SCD within the National Guard Hospital Pediatric-Hematology clinics in Jeddah, Saudi Arabia. SCD, a group of hemoglobinopathies, is recognized as burdensome to parents due to its numerous complications (1). Therefore, our study assesses the barriers that prevent caregivers from seeking HSCT as a curative therapy option that would aid in the relief of these burdens.

In our study, we have employed a structured survey distributed to patient's caregivers that have shown that most of the caregivers of pediatric SCD patients had some background knowledge of HSCT and an overall interest in considering it as a treatment option. However, as much as they favored the pursuit of HSCT, we found some reluctance due to the side effects accompanying treatment. A perfect example is the concern regarding infertility, as HSCT may negatively affect fertility potential. It has been suggested that HSCT can lead to ovarian dysfunction and reduced sperm count. However, the long-term effects of HSCT on fertility are still not fully understood, and more research is needed in this area, especially with the availability of a less toxic conditioning regimen (19, 20). Fertility preservation options prior to HSCT in children with SCD are available, including ovarian tissue cryopreservation and gonadotropin-releasing hormone (GnRH) analogs to preserve testicular function in men and sperm cryopreservation (21). Unfortunately, access to fertility preservation for children undergoing HSCT in our country is very limited and costly. Nonetheless, our team advocates for such options to be more available for this population, and the decision should be made in consultation with the patients' healthcare provider, considering each cases' uniqueness.

One goal of this study is to elicit the treatment misconceptions. Consecutively, we found that many of the patients' caregivers reported optimistic expectations regarding the course of the children's illness. In addition, many believed that the patient's quality of life would improve over time. Nonetheless, we also found that misconceptions related to the nature of the procedure or myths associated with HSCT were roughly scarce. This finding is consistent with the observation by Roth et al., in which most caregivers thought that their children's disease would not limit their life span or hinder them from accomplishing their life goals (22). This could impact the willingness to accept the HSCT.

Although some studies suggested that a lack of understanding of SCD complications could limit the adoption of HSCT as a treatment option, this study found a significant increase in the percentage of caregivers following through with the HLA-typing procedure when recommended by their physician. This fact emphasizes a successful physician-patient relationship that is profound and trusting. We observed that physician's style of counseling patients and families about HSCT as a curative option in Saudi Arabia is variable. Some physicians regularly discuss it; others never address it unless the family approaches the subject, while some are still not very supportive of it, especially for young patients or those with mild phenotypes. Part of that is related to a lack of updated knowledge, unavailability of HSCT service, general pediatricians managing SCD in some centers, and a large proportion of SCD-associated morbidity and mortality occurring in adulthood. Even for clinicians, this gives a false impression that SCD is a livable condition while it is a devastating illness. How healthcare providers in Saudi Arabia discuss SCD prognosis with the family is comparable to what has been reported in the literature (23, 24). In addition, the best methods of communicating disease outcome and treatment options are still understudied in pediatrics and that call for the development of further recommendation for general pediatrician/pediatric hematologist.

To the best of our knowledge, this study is the first to directly assess pediatric patient caregivers' perception of HSCT in Saudi Arabia and the factors encouraging or discouraging them from seeking such treatment. Previous international studies by Michael Roth et al. and Kodish et al. showed similarities in assessing health-related quality of life and disease-concerned factors affecting health which are associated with caregiver's interest in treatment. Hereby, the severity of complications played a significant role in confirming the interest appeal of seeking HSCT treatment (22, 25). Moreover, many have revealed concerns about developing cancer, comorbidities, or mortality with treatment.

It was found that the higher the number of existing sibling-HLA matches, the more the caregivers were likely to be interested in choosing HSCT. It was also noted that the higher the number of children presents with SCD in each family, the more likely the consideration of HSCT. In addition, given the burden of crises associated with SCD, caregivers facing with higher numbers of crises per year were more likely to approve of this procedure in hopes of easing the disease burden, which also applies to the number of absence days of both parent and child. Another finding was that socioeconomic status and the caregiver's education were not factors hindering treatment.

In summary, because of the vast prevalence of SCD in Saudi Arabia and its immense burden not only on those living with it but on the healthcare system, too, we decided to conduct this study to assess the perception of the caregivers of SCD children on HSCT to fill in the gaps of the knowledge of medical practitioners regarding the public opinion on this procedure and their misconceptions toward it in terms of definition, expectations, and concerns. The results of this study were consistent with the fact that most caregivers had enormous trust in their physician's opinions and would follow along with HSCT if it seemed to be fit. However, as our study is the first of its kind in the region, we believe that this calls for a great need to conduct further research in the kingdom on the perception of HSCT. Nonetheless, assessing the patient's and caregiver's current knowledge, readiness, and possible barriers to learning is essential to plan effective ways to deliver patient education, including one-on-one teaching, demonstrations, and a mixed media approach.

## Strengths and limitations

The strengths of this study include an insight into HSCT perceptions that is the first of its kind in the Western region. The strength is that our study will aid in bridging the gap between the lack of awareness among physicians and their patients' caregivers by providing them with our study's analysis and results, reflecting upon the barriers to following through with HSCT, which would be used as a guide for future studies and management approaches of holistic aspects. Some limitations regarding the sample size being relatively small and it was done in a single center.

## Data Availability

The datasets presented in this article are not readily available because the data are not publicly available due to privacy or ethical restrictions. Requests to access the datasets should be directed to dania_monagel@hotmail.com.
