# Supplementary material for: The perception of pediatric sickle cell anemia patient's caregivers toward hematopoietic stem cell transplantation (single-center experience, Saudi Arabia)
Source: Front Pediatr. 2023 May 23;11:1205351. doi: 10.3389/fped.2023.1205351 (PMC10242087; doi:10.3389/fped.2023.1205351)
Supplement: Supplementary file 1 [file Datasheet1.pdf]

This study will be a cross-sectional survey in which approximately 100 sickle cell disease patients' caretakers will be subject to our survey's data collection. Our study subjects will be briefly interviewed and surveyed utilizing an online questionnaire provided to them via an electronic tablet device, and consent will be taken before data collection. Moreover, data collected will be statistically analyzed using Statistical Product and Service Solutions (SPSS), a software analysis system. An Arabic version will be made in case if we decide to distribute it on the patient.

Parent's demographic information :

1- Agree
2- Disagree

1-How old are you?
[number/range]

2-What is your gender?
a- Male
b- Female

3-What is your highest level of education?
a- Elementary school.
b- Middle School.
c- High School Graduated
d- College Graduated
e- School Degree Graduated

4- what is your marital status?
a- Married
b- Single
c- Widowed
d- Divorced

5- what is your occupational status?

a- Employee
b- Student
c- Non
d- Other

6- What is your relationship with the child?
a- Father
b- mother
c- Relatives
d- Other

Child’s Demographic information :

7- what is your child’s gender?
a- Male
b- female

8- How old is your child?
[number]

Information about the disease:

9 -What is the type of sickle cell disease your child has?
a- Hemoglobin SS
b- Hemoglobin SC
c- Hemoglobin Sickle Beta Thalassemia
d- Other……
e- Don’t know.

10-How many crises has your child had in the past year?
a- 0
b- 1-2
c- 3-4
d- 5-6
e- 7 or more

11-How many days of school does your child miss each year because of his/her sickle cell disease?(before corona)
a- Non
b- 1-3
c- 4-6

d- 7-10
e- More than 10.

12-How many days of work do you miss each year because of your child’s sickle cell disease? (before corona)

a- None
b- 1-3
c- 4-6
d- 7-10
e- More than 10

13- Do you have other children with sickle cell disease? If yes How many?

a- Yes
b- No

[number]

14-Has your child ever had any of the following complications:

Complication	Yes	No	Don’t know
Bone necrosis			
Stroke			
Abnormal Trans-cranial Doppler (TCD)			
Sickle cell lung disease			
Sickle cell kidney disease			
Sickle cell liver disease			
Sickle cell related heart disease			
Bone infection (osteomyelitis)			
Acute chest syndrome			
Pain crisis			
Splenic sequestration			
Surgery to remove spleen or gall bladder			
Delayed puberty and reduced growth			
Chronic leg ulcer			

15-Has your child ever received any of the following treatment for sickle cell disease:

Treatment	Yes	No	Don’t know
Hydroxyurea			
Simple blood transfusion			
Exchange transfusion			

Penicillin prophylactic			

16- Do you believe your child's sickle cell disease will get better, worse, or stay the same when he/she is older?

a. Better
b. Worse
c. The Same

17-Have your child been offered to have (HLA typing) a process of finding a family match for possible stem cell transplant in the future?

a. Yes. If Yes: Number of matches in the family: …..,
b. No.

18- Have you heard/read about stem cell transplant as curative option for sickle cell disease?

a. Yes. Source of info:……, The doctor Name at heme clinic:…..
b. No.

<u>If the answer is yes for question 18 then proceed with the following questions:</u>

19- Would you like more information about stem cell transplant for sickle cell disease?

a. Yes. Source of info:……, The doctor Name at heme clinic:…..
b. No.

20-What is a sibling-matched stem cell transplant for sickle cell?

a. A patient with sickle cell disease donates stem cells to a brother or sister.
b. A patient with sickle cell disease donates stem cells to another person with sickle cell disease.
c. A patient with sickle cell disease gets healthy stem cells from a brother or sister to replace abnormal blood cells in the patient's body.
d. A patient with sickle cell disease gets healthy stem cells from someone not related to them to replace abnormal blood cells in the patient's body.

For the next questions, please choose from 1-5 notice the following:

1= Definitely NOT (0%)
2= Probably NOT (25% chance)
3=Maybe (50% chance)
4=Probably (75% chance)
5=Definitely (100% chance)

21- Do you believe your child’s sickle cell disease will prevent your child from achieving certain life goals?

	1	2	3	4	5	
😒	○	○	○	○	○	😀

22-If your doctor recommended a stem cell transplant for your child, and you have another child who is a match, how likely would you be to go for the stem cell transplantation?

	1	2	3	4	5	
😒	○	○	○	○	○	😀

23-What reason MOST explains why you would not be likely to go for stem cell transplantation?

a. I do not have enough information about stem cell marrow transplant to decide
b. I would be concerned about the risks and possible side effects
c. I do not think it would benefit my child
d. I do not think my child needs a stem cell transplant
e. None

24-Which of the following possible side effects of stem cell transplant are you most concerned about?

a. Dying
b. Graft versus Host disease
c. Not being able to have children (infertility).
d. Risk of secondary malignancy.
e. None.

25- What is the risk of dying from stem cell transplant?

a. 0%
b. 5-10%
c. 11-30%
d. >30%

26- What percentage of children with sickle cell disease is cured after stem cell transplant?

a. 0 %
b. 1-20 %
c. 50 %
d. 80-90 %
e. 100 %

__المعلومات الديموغرافية للأهل__

1-العمر؟

[العدد]

2- الجنس؟

أ-ذكر

ب- انثى

3-ما هو أعلى مستوى تعليمي لديك؟

أ- المدرسة الابتدائية.

ب- المرحلة المتوسطة.

ج- الثانوية العامة

د- خريج جامعة

هـ- الدرجة الجامعية المتخرجين

4- ما هي حالتك الاجتماعية؟

أ- متزوج

ب- أعزب

ج- أرمل

د- مطلق

5- ما هو وضعك المهني؟

أ- موظف

ب- طالب
ج- غير ذلك

6- ما علاقتك بالطفل؟
أ- الأب
ب- الأم
ج- أخرى ... ...

<u>المعلومات الديموغرافية للطفل:</u>

7- ما هو جنس طفلك؟
أ -ذكر
ب- أنثى

8- كم عمر طفلك؟
[رقم]

معلومات عن المرض:

9- ما هو نوع مرض فقر الدم المنجلي الذي يعاني منه طفلك؟
أ- الهيموجلوبين SS
ب- الهيموجلوبين SC
ج- الهيموجلوبين المنجلي بيتا ثلاسيميا
د- أخرى ......
ه- لا أعرف.

10-كم عدد الأزمات التي مر بها طفلك في العام الماضي؟
أ- 0
ب- 1-2

ج- 3-4

د- 4-5

هـ 6 أو أكثر

11- كم يوم دراسي يتغيب عنه طفلك كل عام بسبب مرض فقر الدم المنجلي؟

أ- لا

ب- 1-3

ج- 4-6

د- 7-10

هـ أكثر من 10.

12-كم يوم عمل تتغيب كل عام بسبب مرض فقر الدم المنجلي لدى طفلك؟

أ- لا شيء

ب- 1-3

ج- 4-6

د- 7-10

هـ أكثر من 10

13- هل لديك أطفال آخرون يعانون من مرض فقر الدم المنجلي؟ إذا كانت الإجابة بنعم ، فكم عددهم؟

أ- نعم

ب- لا

[رقم]
